# Recent Advances in Properties and Applications of Carbon Fiber-Reinforced Smart Cement-Based Composites

**DOI:** 10.3390/ma16072552

**Published:** 2023-03-23

**Authors:** Yali Hao, Cheng Shi, Zhenxiao Bi, Ziqiang Lai, Anming She, Wu Yao

**Affiliations:** 1 School of Materials Science and Engineering, Tongji University, Shanghai 201804, China; 2 Key Laboratory of Advanced Civil Engineering Materials of Ministry of Education, Tongji University, Shanghai 201804, China

**Keywords:** carbon fiber-reinforced cement-based composites (CFRCs), enhancement and modification mechanism, mechanical properties, durability, smart properties, application

## Abstract

Under the strategies of low-carbon and environmental protection, promoting green technology innovation to achieve carbon neutrality in the construction field has become a universal goal. As the building material with the highest consumption, concrete has gradually begun to transform into a multi-functional and intelligent product. Therefore, the research on carbon fiber-reinforced cement-based composites (CFRCs) is of relative interest. It mainly uses carbon fibers (CFs) with high elasticity, strength, and conductivity to disperse evenly into the concrete as a functional filler, to achieve the intelligent integration of concrete structures and function innovatively. Furthermore, the electrical conductivity of CFRC is not only related to the content of CFs and environmental factors but also largely depends on the uniform dispersion and the interfacial bonding strength of CFs in cement paste. This work systematically presents a review of the current research status of the enhancement and modification mechanism of CFRC and the evaluation methods of CF dispersion. Moreover, it further discusses the improvement effects of different strengthening mechanisms on the mechanical properties, durability, and smart properties (thermoelectric effect, electrothermal effect, strain-sensitive effect) of CFRC, as well as the application feasibility of CFRC in structural real-time health monitoring, thermal energy harvesting, intelligent deformation adjustment, and other fields. Furthermore, this paper summarizes the problems and challenges faced in the efficient and large-scale applications of CFRCs in civil engineering structures, and accordingly promotes some proposals for future research.

## 1. Introduction

With the continuous development of high-strength and high-performance technologies in civil engineering, concrete has been widely used in large-scale structures and infrastructural engineering because of its low cost and stable performance [1,2]. However, the problems of traditional concrete are becoming increasingly prominent (single function, low tensile strength, and easy fracture), which seriously restrict the development of concrete materials [3,4]. Moreover, under the actions of adverse factors such as environmental changes, external loads, and construction, concrete structures inevitably produce damage accumulation and resistance attenuation damage and even lead to catastrophic accidents [5]. Therefore, the vulnerable parts of the structure should possess multi-functions (real-time health monitoring [6], self-regulation [7], self-protection [8], and so on), such that as a result, the structure can display early warning signs before major disaster events, which is also a research hotspot for scholars around the world.

To prepare an intelligent, cement-based, material-integrating structural function and multiple functions, smart-sensing materials such as a fiber Bragg grating sensor [9], shape memory alloy [10], and lead zirconate titanate piezoelectric ceramics [11,12] are usually embedded in concrete for real-time monitoring of stress, strain, damage, and temperature changes. However, due to the characteristics of a high cost, low durability, and poor compatibility with the concrete matrix, such sensors bear great environmental pressure from preparation to recovery [13]. Accordingly, to further improve the environmental compatibility of cement-based materials and reduce material costs, the latest research focuses on innovatively adding some conductive fillers to cement-based materials, such as carbon nanotubes [14,15], graphene oxide [16], carbon fibers (CFs) [17], and iron oxide nanoparticles [18]. Numerous studies have [19,20] shown that these conductive fillers promote the generation of cement hydration product C-S-H gel through nucleation to further refine the internal pore structure, to achieve the purpose of improving the mechanical properties and durability of the structure. Simultaneously, cement-based materials are also endowed with superior electrical, thermal, electromagnetic, and other sensing functions owing to their excellent inherent performance. Therefore, an enormous amount of research effort has been put into studying carbon fiber-reinforced cement-based composites (CFRCs). CFs, as a multifunctional filler, are uniformly dispersed into cement paste. On the one hand, based on their characteristics of a light weight and high strength, CFs have a great highly technical attraction in strengthening and toughening [21], reducing drying shrinkage [22], and other mechanical properties of concrete. On the other hand, CFs possess a thermal and electrical conductivity similar to metal, thermostability and corrosion resistance similar to ceramic, and the softness and knittability of textile fibers apart from their inherent characteristics. Under the action of a load, temperature, and other external factors, CFRC will present pressure [23] and temperature sensitivity [24] with a change in its CF spacing, which is one of its most unique characteristics. Hence, as a functional filler, CFs have prominent advantages in the preparation of smart concrete [25].

As the multi-functionalization and intellectualization of concrete structures has become possible, CFRC is just the right kind of smart material widely used to meet the needs of the intelligent development of civil engineering. Moreover, it can realize the self-diagnosis, self-regulation, and self-protection of concrete structural damage within the real service environment and during natural disasters, which is of great significance to reducing economic losses. Through the unremitting efforts of scholars at home and abroad, the conductive properties of CFRC and its influencing factors have been studied deeply. However, there are some uncertainties and discreteness in the smart properties of CFRC due to the existence of hydrophobic CFs with some defects, including a poor dispersion and weak bonding strength between the CFs and concrete matrix, which dominantly restrict the engineering application of CFRC. The novelty of this paper is that it provides a summary of the latest research progress of the applications in the field of smart CFRC. Contraposing the difficulty of CF dispersion and weak interfacial adhesion, an up-to-date summary of the reinforcement and modification methods used in different studies is given first. The influence of the conductive transmission mechanism of CFRC is furthermore given along with a discussion in this review. Additionally, based on the available limited literature, the improvement effects of the mechanical properties, durability, and smart properties (electrothermal, thermoelectric, and sensing effects) of CFRC under different modification and strengthening mechanisms are systematically analyzed. Special attention is also paid to the potential applications of CFRC in the fields of structural real-time health monitoring, thermal energy harvesting, and intelligent deformation adjustment. Finally, the critical problems that need to be solved urgently are pointed out, with the purpose of providing a useful reference for further research on CFRC.

## 2. Review Objective and Methodology

### 2.1. Review Objective

Over the course of the systematic literature review of CFRC, it was found that the incorporation of CFs can not only significantly enhance the strength and toughness of CFRC but also improve its electrical conductivity, with the intrinsic functions of self-sensing and self-adjusting. Considering these characteristics, on the one hand, CFRC can act as a sensor to reflect its degree of internal damage. On the other hand, it can be used as a driver to adjust temperature, stress, and deformation, which has attracted much attention in the research and development of intelligent material structure systems. To improve the application potential of CFRC in smart structures, it was imperative to systematically summarize the current research progress of CFRC and identify the prevailing achievements to compile the existing research gaps for further research. Researchers worldwide have focused on various objectives during their investigations of CFRC, leading to the results obtained being somewhat discrete. Thus, the compilation of the existing research gaps based on a critical combination of existing research findings is essential to provide a broad perspective of the current research level. In this work, while attempting to review the basic and pioneering progress of CFRC research work by domestic and foreign scholars, we summarize the potential drawbacks that may exist in CFRC from its preparation to application, such as the non-uniform dispersion of CFs and poor interfacial bond strength. Moreover, the significance of this review is further elaborated in terms of the enhancement potential of different enhancement modification mechanisms on the mechanical, durability, and smart properties of CFRC.

### 2.2. Methodology

This review further summarizes the existing research progress on the mechanical, durability, and smart properties of CFRC by collecting and evaluating the related literature published previously. Regarding data collection, it was mainly realized using the well-known literature database sources such as Web of Science, SCOPUS, and Google Scholar. Important strings such as “carbon fiber”, “cement” and so on were typed in the keyword search box and separated with commas to retrieve the literature related to CFRC. The number of articles to be included in this review was then further narrowed down precisely by title, abstract, and keywords in the preliminary assortment of the literature data. Moreover, irrelevant document types were excluded through continuous optimization to ensure that the documents included corresponded to the topic being explored. Finally, all research articles retrieved from each source database were imported into Mendeley for their literature management and intelligent identification to eliminate duplicates in the research paper database. CFRC is a developing field that has received a lot of attention from researchers since its inception. Through this technique, a more comprehensive scope of relevant information about the topic was obtained. Whereas the complete literature search list on CFRC is about 4000, this review is summarized based on these references.

### 2.3. Data Collection and Categorization

**Figure 1:**
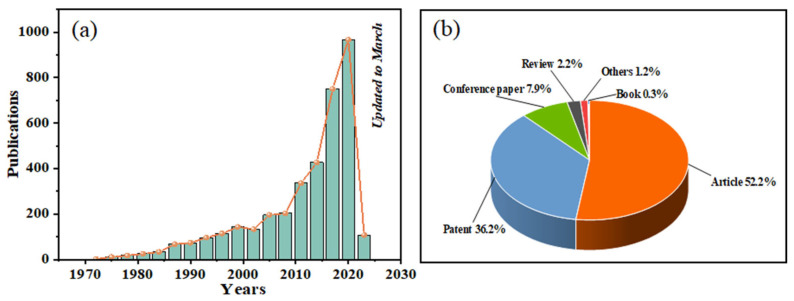
(**a**) Publication trend of the CFRC literature; (**b**) Types of documents in the CFRC literature.

The comprehensive review of the current research progress on CFRC from its preparation to properties to application was conducted based on both scientometric and traditional analytical methodologies. Therein, the scientometric analysis method is essential for data collection and classification, as it can provide reliable, reasonable, and relatively less subjective results for the assessment of the characteristics of the available retrieved data. As presented in Figure 1, the scientometric analysis of the trends and types of the CFRC literature was conducted based on the large Web of Science database in this paper. The research on CFRC dates back several decades but the publication of the related literature remained slow when the concept of CFRC was first introduced. By 2010, the publication speed of the CFRC literature was accelerated, and the period from 2016 to 2020 was the most fruitful period, with the annual number of published papers increasing exponentially (Figure 1a). The reason for this phenomenon is the increasing demand for the intelligent development of structures driven by advances in science and technology. The scientometric statistical results of the types of documents searched for related to CFRC are presented in Figure 1b. As can be seen from the results of the article types, the journal literature, patents, and conference papers account for 52.2%, 36.2%, and 7.9%, respectively, which constitute an important part of the source of the CFRC database. The rest consists of other sections such as reviews and books, which account for about 3.7% of the overall CFRC literature database.

## 3. Reinforcement and Modification Mechanism of CFRC

**Figure 2:**
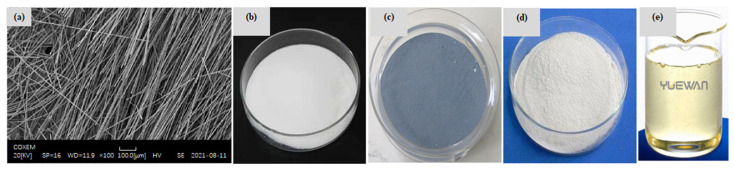
Morphology of (**a**) CFs, (**b**) water-reducing agent, (**c**) silica fume, (**d**) dispersant, and (**e**) defoamer ((**a**) are from Ref. [28] while (**b**–**e**) are proposed by this paper).

It is widely accepted that CFs have a carbon content of more than 90% and are endowed with high tensile strength, high elastic modulus, and good electrical conductivity (Figure 2a). Generally speaking, CFs are divided into three types, namely polyacrylonitrile-based CFs, pitch-based CFs, and metal-plated CFs, and their resistivity ranges from 1 × 10^−5^ to 1 × 10^−3^ Ω·cm. Among these fibers, the conductivity of polyacrylonitrile-based CFs and metal-plated CFs is relatively good; yet, the high cost of metal-plated CFs cannot be ignored. Hence, polyacrylonitrile-based CFs are considered a cost-effective conductive material under the premise of a comprehensive consideration of their resistivity, cost, dispersibility, and durability. However, due to the hydrophobicity and light weight of CFs, the main challenge faced by CFRC is the poor interfacial bonding strength between CFs and the cement matrix. Additionally, a series of defects are introduced by fibrous agglomeration. Research has established that not only is the potential of CFRC in terms of its mechanical properties not being fully utilized [26], but its conductivity is also affected [27]. Thus, it is necessary to improve CFRC through appropriate reinforcement and modification processes to obtain good performance.

### 3.1. Modification of CFs

**Figure 3:**
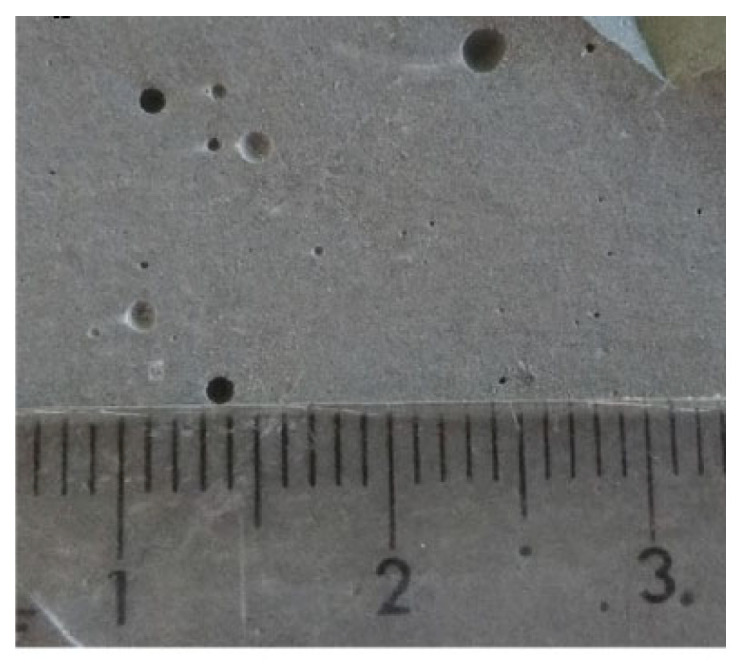
Macroscopic optical image of the CFRC including dispersant [34].

**Figure 4:**
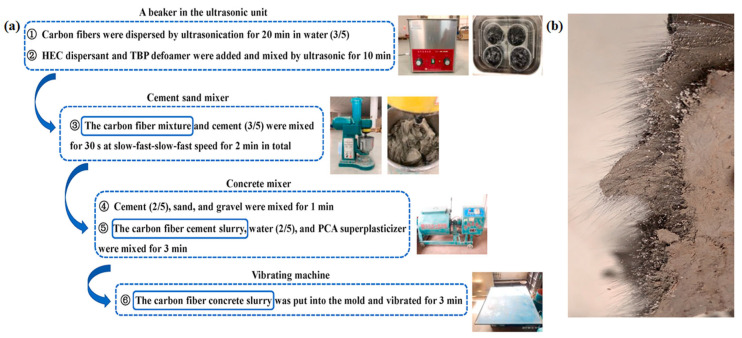
(**a**) Dispersion and mixing process of CFRC; (**b**) Fracture section [33].

Extensive efforts have been made to improve the degree of dispersion of chopped CFs in the cement matrix, which is mainly divided by mechanical separation and chemical treatment. At present, ultrasonic [29], ball milling, and high-shear mixing technologies are frequently used for the simple mechanical separation of CFs, and then the surface of CFs is further modified by dispersing [30] and water-reducing agents (Figure 2b) [31]. This is mainly based on the principle that six-membered rings and polar hydroxyl groups in the dispersant unit structure form hydrogen bonds with polar groups and polar water molecules on the surface of CFs to increase the hydrophilicity and wettability of CFs, thus promoting the dispersion of CFs into single filaments [29]. The effect produced by the improved dispersion of CFs can be used to explain the formation of finer and denser microstructures of CFRC. Although the dispersant can effectively reduce the open porosity, there is no way to avoid the dispersant generating large voids during the specimen preparation stage. Furthermore, these pores are far beyond the measurement range of the MIP technique, which is extremely detrimental to the mechanical properties of CFRCs (Figure 3). Considering that silica fume is a mineral admixture with fine particles and a spherical distribution (Figure 2c), Wen et al. [32] found that the combined action of the dispersant methylcellulose (Figure 2d) and silica fume was fairly effective in promoting the dispersion of CFs and reducing porosity and electrical resistivity. Meanwhile, Wang et al. [33] used a modified molding process combining chemical dispersion and multi-level physical processes to treat 20 mm long CFs and then prepare CFRC (Figure 4a). As can be seen from its fracture surface, this process can obtain a CFRC with evenly distributed CFs (Figure 4b).

**Figure 5:**
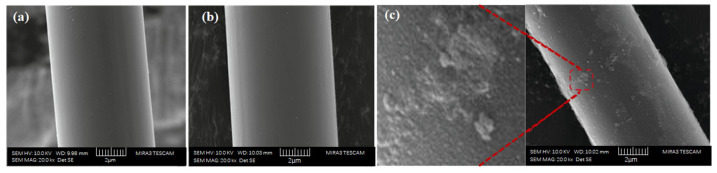
(**a**) Ordinary CFs; (**b**) CFs treated with nitric acid; (**c**) Nano-silica coated on the surface of CFs [41].

The surface of CFs is smooth and free of active groups, and the bonding between CFs and cement mainly depends on the attractive van der Waals forces. Based on the uniform dispersion of CFs, its surface can also be activated by plasma treatment [35], in situ growth [36], or chemical oxidation [37] to improve the interfacial bonding strength. Kim et al. [35] found that CFs treated with argon, nitrogen, or oxygen plasma could increase their number of hydrophilic functional groups on the surface, accelerate the hydration of cement, and make the cement matrix around the CFs more compact. Meanwhile, Li et al. [38] reported that the nano-silica with a negative charge could be uniformly dispersed on the surface of CFs employing electrophoretic deposition. The specific surface area increased by in situ growth modification effectively improves the dispersion and interfacial bonding strength of CFs when the chemical bonding and infiltration conditions of CFs are similar [36]. Additionally, Fu et al. [39,40] confirmed that a great quantity of hydrophilic oxygen-containing functional groups could be formed in strong oxidants, thus providing more binding sites for SiO_2_ and effectively improving the dispersion ability of CFs. Figure 5 shows the micromorphology of CFs before and after modification with the silane coupling agent. As seen in the figure, untreated CFs have a clean surface and regular longitudinal grooves (Figure 5a). After being soaked in nitric acid, their surface activity is enhanced, and it presents a string shape in the axial direction, thus increasing its surface roughness (Figure 5b). After immersion in a hydrophilic SiO_2_ coating solution for 3 h, a large number of nano-SiO_2_ particles will grow on the CF surface (Figure 5c). Therefore, the interfacial bonding strength can be effectively improved by stimulating the reaction of nano-SiO_2_ on the CF surface with cement hydration products.

### 3.2. Optimization of CFRC Mixing Process

Whether CFs can be uniformly dispersed in the stirring process is still a crucial issue in the preparation of CFRC. The purpose of mixing is not only to strengthen the chemical reaction of the mixture and accelerate the heat transfer rate of the mixture but also to consider the uniform dispersion of CFs in the cement matrix and the minimum breaking loss. Wu et al. [42] examined the resistivity changes of CFRC that were mixed by the wet mixing process and the dry mixing process, respectively, and the results indicated that under the same content of CFs, the wet mixing process can achieve a relatively uniform dispersion of CFs and is not easy to aggregate immediately. Additionally, the conductive property of CFRC prepared by the wet mixing process is better than that of the dry mixing process. Yang et al. [43] evaluated the effect of mixing time and throwing order on the dispersion of CFs based on the dispersion coefficient and variation index. The results showed that CFs could be evenly dispersed in the slurry by mingling it in water first and then adding cement to stir for 2 min.

Although the strengthening and modification process of CFRC has brought positive benefits in elevating mechanical properties, the commonly used modification technologies still come with defects such as a low dispersion efficiency, poor dispersibility, and difficulty in long-term storage. In brief, the uniform dispersion of CFs in cementitious systems is still a challenging problem, so it is imperative to seek a simpler, faster, and lower energy consumption preparation process.

### 3.3. Improvement of CF Dispersion Evaluation Mechanism

**Figure 6:**
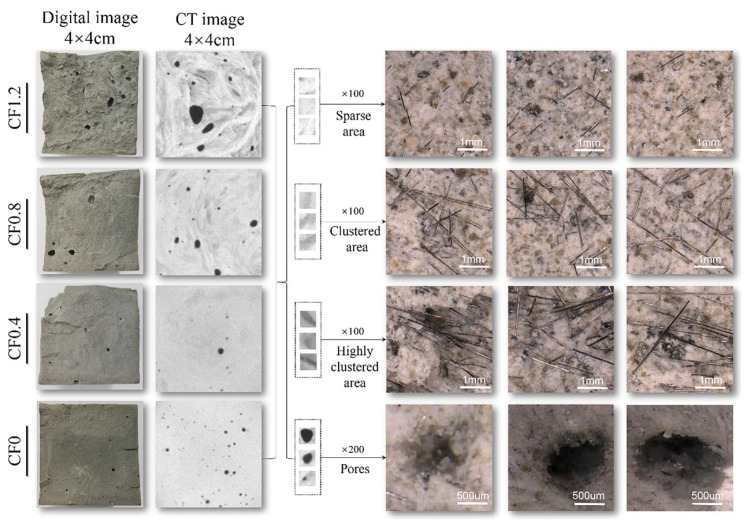
The actual fracture appearance, CT images, and micrograph of CFRC [46].

The uniform distribution of CFs is one of the primary prerequisites for improving the mechanical and electrical properties of CFRC. At present, the method of CF dispersion evaluation in cement matrix is mainly divided into direct and indirect evaluation. The method of direct evaluation is mainly used to qualitatively evaluate the dispersion of CFs by measuring the resistivity of hardened cement paste or fresh cement paste under the action of alternating current, which is fast, convenient, and relatively economical. Woo et al. [44] characterized the dispersion degree of CFs in CFRC based on AC impedance spectroscopy, and comprehensively analyzed the orientation, overall segregation, and local agglomeration of CFs in the cement matrix combined with the point probe technique and dispersion factor. Zhu et al. [29] indirectly evaluated the dispersion of CFRC according to the quality variation coefficient, SEM, XRD, BET, and other indicators. Yuan [45] proposed a new method of deep learning for evaluating the distribution of CFs based on SEM, which mainly extracted the components of CFs from the scanned image and then made a detailed analysis. Meanwhile, Gao et al. [46] discussed the influence mechanism of CF distribution on CFRC performance utilizing computer tomography and gray entropy correlation analysis. This method is more intuitive and applicable for the evaluation of a small range CF dispersion state and can obtain the true CF distribution at the microscopic level of CFRC, as shown in Figure 6. Based on the existing evaluation systems, it is necessary to make further improvements to effectively reflect the dispersion state of CFs in cement paste. This will better guide the design of CFRC preparation technology and promote its application in advanced structural materials.

## 4. Conductive Mechanism of CFRC

**Figure 7:**
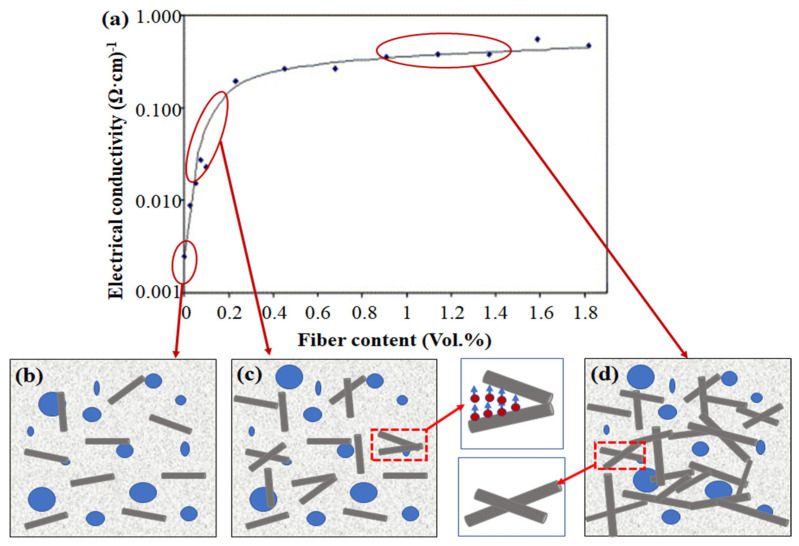
(**a**) Variation trend of electrical conductivity versus CF content [49] and (**b**–**d**) Conductive mechanism diagram of CFRC proposed by this paper.

The extensive carbon net plane formed by aromatic rings in CFs is a conjugated system and the movement of π electrons is an important factor for the conductivity of CFRC. Numerous studies have shown that the conductivity of the CFRC multiphase system is mainly based on electrons and holes, and it is also affected by ionic conductivity [47]. The Ca^2+^, K^+^, Na^+^, and OH^−^ in the pore solution will directionally move to the CF surface to form a conductive water film under the action of an applied electric field. The ionic conductivity process is also closely related to the concentration of conductive ions in the pore fluid, the moisture content, and the hydration degree [48]. The ionic conductivity of specimens with a 28 d curing age and drying treatment can be neglected. Moreover, electronic conduction can be divided into the tunnel conduction effect and contact conduction effect, which are mainly affected by the content of CFs [49,50] (Figure 7a). When the content of CFs is small, the distance between adjacent CFs is greatly enlarged for π electrons to pass through the potential barrier, and a reduction in resistivity is unobvious (Figure 7b). With the gradual increase in CF content, the distance between adjacent CFs is greatly reduced. Generally, when it is less than 2.5 nm, π electrons can pass through the potential barrier between two adjacent CFs to form a conductive path through the tunneling conduction effect (Figure 7c). This phenomenon mainly occurs near the percolation threshold, which plays a decisive role in determining the overall resistivity of CFRC. When surpassing a certain amount, a large number of chopped CFs are overlapped with each other to generate contact conductivity and a relatively stable resistivity can be obtained (Figure 7d) [27,51,52]. Besides the content of CFs, the aspect ratio of CFs, internal pore structure, and the interfacial bonding strength between the matrix and CFs are other crucial factors defining the conductivity of CFRC. Celzard et al. [53] analyzed the influence mechanism of the CF aspect ratio on the percolation threshold and found that the larger the CF aspect ratio, the smaller the percolation threshold. According to Wu et al. [54] as well, the percolation threshold of CFRC is closely related to the dispersion of CFs. Meanwhile, the experimental results of Chung et al. [55] pointed out that the closure and development of internal cracks and the interface contact state of CFs and cement-based composites are also indispensable factors affecting the conductivity of CFRC.

In brief, the conductive mechanism of CFRC is extremely complex and these conduction mechanisms coexist and relate with each other. Although the above research could explain the sudden change in the conductivity of CFRC near the percolation threshold, each model derivation has specific applicable conditions. Thus, it is worthwhile to propose a more general, simple, and accurate CFRC conductivity model for future investigation.

## 5. Property Evaluation of CFRC

### 5.1. Mechanical Properties

**Figure 8:**
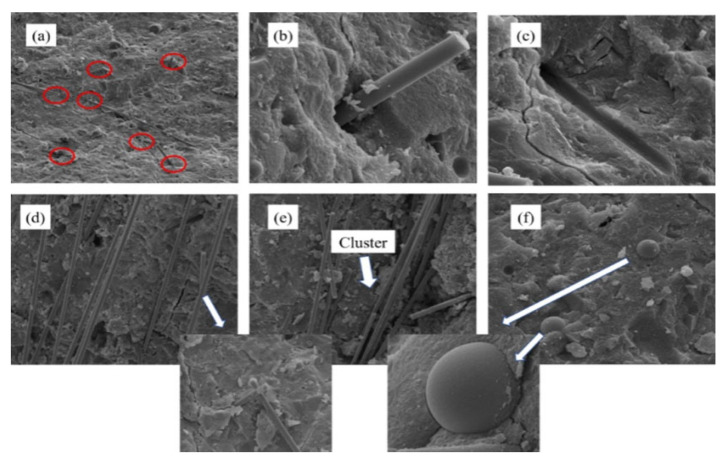
Morphology of CFRC at (**a**–**c**) low CF content and (**d**–**f**) high CF content [29].

Owing to the abundant inherent properties of CFs, good dispersion could play a significant role in preventing the cracking and toughening of CFRC [56,57]. This could be attributed to the fact that doped CFs can optimize the internal pore structure on the microscopic level, effectively bridging the two ends of the crack and delaying the further formation and development of the crack, which has been assigned to be the strengthening effect on improving the mechanical properties of CFRC (Figure 8a–c) [58]. However, when the fraction of CFs exceeds a certain amount, CF addition can introduce internal defects due to uneven dispersion. It furthermore leads to the stress concentration of the CFRC under stress, causing the efficiency of the reinforcing phase not to be fully played out (Figure 8d–f). Moreover, the surface hydrophobicity of CFs as well might cause damage to the interfacial bonding strength between CFs and the cement matrix, resulting in a weakening effect on the mechanics and durability of CFRC [19]. Within CFRC, these two mechanisms restrict and develop each other, and their influence on performance is mainly manifested in the different dominant positions of the two mechanisms. It is indicated that the weakening effect introduced by CF addition should be taken into due consideration and an appropriate reinforcement and modification technology should be further sought out to obtain a good mechanical property.

Wang et al. [59] reported that CFs can obtain a uniform dispersion in the new dispersant hydroxyethyl cellulose solution with the assistance of ultrasonic technology. A certain amount of defoamer (Figure 2e) can effectively reduce the bubbles in cement paste and improve its fluidity. The compressive strength, tensile strength, and elastic modulus of CFRC prepared by this method are, respectively, 20%, 140%, and 26.8% higher than specimens without CFs. The mechanical properties of CFRC are well related to the interfacial bonding strength between CFs and cement paste. Heo et al. [41,60] designed a unique treatment method that can uniformly coat nano-SiO_2_ particles on the surface of CFs for hydrophilic modification based on the chemical reaction of a silane coupling agent. It was concluded that the significant improvement in the chemical binding force of the interface transition zone could be achieved through the C-S-H gel formed by the reaction of nano-SiO_2_ and Ca (OH)_2._ Furthermore, the CFRC modified by nano-SiO_2_ coating increased the energy consumption during drawing failure, which contributed to the deflection and blocking of crack diffusion. Compared with ordinary mortar, the frictional bond strength and bending strength were increased by 27.3–33.0% and 58.1%, respectively. In contrast, the compressive strength decreased somewhat. This might have been caused by the air content introduced by the increased volume fraction of CFs [61]. Yet, Li et al. [38] introduced a new modified mixing method, in which nano-SiO_2_ was deposited on the surface of CFs uniformly by electrophoresis. The shear bond strength and total fiber drawing work of CFRC were increased by 93% and 182%, respectively. This confirmed the great potential of electrophoresis in improving the mechanical properties of CFRC. Similarly, ozone treatment [40] and plasma modification [35] can also improve the surface roughness and hydrophilicity of CFs. Apart from the modification of CFs, mixing CFs with other fibers can also modify the microstructure of CFRC. Yao et al. [62] stated that the mixing of CF steel fibers was more beneficial to improving the mechanical properties of CFRC than that of CF polypropylene fibers, which could increase the compressive strength, cleavage strength, and breaking modulus by 31.4%, 36.5%, and 32.9%, respectively. The reason for this phenomenon is that polypropylene fibers belong to low modulus fibers, while steel fibers have the outstanding advantages of having a high elastic modulus and flexural toughness, so the hybridization of CFs and steel fibers can give full play to their respective performance advantages. Moreover, silica fume particles are fine, which can fill the gap well between cement particles and aggregate within the holes introduced by the addition of CFs, such that the integrity of CFRC is better and the compactness is higher. Meanwhile, a significant improvement in silica fume facilitating the dispersion of CFs can be observed. In brief, appropriate reinforcement modification technology should be considered to improve the dispersion degree of CFs in the cement matrix, enhance the interfacial bond strength, and refine the pore structure, which can achieve the objective of strengthening the mechanical properties of CFRC.

### 5.2. Durability of CFRC

The durability of CFRC in the actual service environment determines its commercialization and application potential in the field of intelligent construction [63]. CFs used in engineering are mostly one-dimensional carbon materials with high aspect ratios, stable chemical properties, and high toughness [64]. The CFs scattered in the concrete can give full play to the bridging effect, which contributes to inhibiting the generation of connecting cracks and improving the anti-expansion capacity of the structure. Consequently, significant benefits can be generated in improving impermeability, frost resistance, sulfate corrosion resistance, carbonation resistance, and chloride ion permeability of structures [65,66,67,68,69]. Yuan et al. [67] evaluated the effect of CF content on the frost resistance durability of CFRC based on the relative dynamic elastic modulus and mass loss rate. It was found that with the increase in CFs, the relative dynamic elastic modulus of CFRC incorporating 1 wt.% CFs could remain at 90% after 150 freezing–thawing cycles, showing excellent frost resistance. According to Guo et al. [70], CFRC as well had superior corrosion resistance in their simulated seawater sea sand concrete environment.

However, CFs added directly cannot effectively fill the pores inside CFRC; on the contrary, it will also introduce a series of defects due to its poor dispersion. It should be pointed out that there still exist possibilities for further amelioration in improving the durability of CFRC. For this reason, extensive attempts have been made to refine the pore structure through a fiber hybrid [71] and add some ultra-fine mineral admixtures [72] or nano conductive fillers [73] to improve the durability of the structure. Zhou et al. [71] found that the average bending strength and splitting tensile strength of CFRC prepared by mixing CFs and polypropylene fibers can reach 8.4 MPa and 3.7 MPa, respectively, and the chloride ion diffusion coefficient was 2.26 × 10^−12^ m^2^/s. Compared with CFRC doped with CFs alone, its anti-fracture performance and durability were greatly improved. Abbasi et al. [72] confirmed that mixing CFs and silica fume into concrete can not only improve the phenomenon of CF agglomeration but also increase the 28 d splitting tensile strength by about 25–38%. The reduction in porosity is also beneficial to improving the resistance of CFRC to chloride ion penetration, sulfate attack, and carbonization. Ashraf [74] designed CFs and silica fume to be used as reinforcing agents to jointly improve the structure of the poor interfacial transition zone of recycled concrete. The results showed that the compressive strength, tensile strength, and bending strength of the recycled concrete at 28 d increased by 20%, 34%, and 46%, respectively, compared with the control group. Moreover, it only had a 6% mass loss under the action of an acid attack, which improved its acid resistance durability by nearly 40%. Furthermore, a large number of research achievements have been made in improving the durability of CFRC by using nanomaterials based on its filling effect, crystal nucleus effect, and surface activity effect [75]. Zuo et al. [76] stated that CFRC containing both CFs and carbon nanotubes showed a more prominent crack-bridging effect, which contributed to restricting the initiation and extension of cracks. Wang [77] reported that nano-CFs could improve the microscopic morphology of CFRC through fiber bridging and pore filling, which could obtain satisfactory durability at a dosage of 0.3%. Similarly, the influence mechanism of carbon black mixed with CFs to enhance the durability of CFRC through a reinforcement network formed by internal interaction cannot be ignored. Moreover, the mechanical and durability properties of CFRC have been reviewed and summarized, and the two typical enhancement categories have also been provided in this work. Detailed information on the mechanical and durability properties is presented in Table 1, Table 2 and Table 3, which contribute to compare the properties of designed mixtures with different ratios.

### 5.3. Smart Properties

#### 5.3.1. Thermoelectric Effect

As a multifunctional composite, apart from its remarkable mechanical properties, Wen et al. [82] found that CFRC possesses good thermoelectric properties. This might be related to the inherent properties of CFs. Most of the fibers used in cement-based materials are polyacrylonitrile-based based CFs and are subjected to a carbonization process with temperatures up to 600–1750 °C in the manufacturing stage, which cause abundant excess holes to remain in the valence band [76]. When there is a temperature gradient across the CFRC specimen, the hole carriers wearing a positive charge will move from the hot end to the cold end of the material, thereby generating a current [83]. With the extensive application of CFRC in the field of civil engineering, researchers have found that its thermoelectric performance could capture the weak energy generated by solar radiation under the action of the temperature gradient and recover and convert it into electric energy. Thus, the thermoelectric technology of CFRC shows tremendous development potential in regulating the surface temperature of buildings in summer and recycling industrial waste heat. Additionally, it also provides new insights into energy conservation and emission reduction in the field of construction [84].

**Figure 9:**
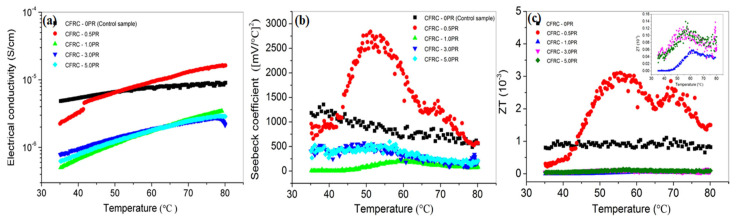
Thermoelectricity of CFRC prepared by treating CFs with different contents of phenolic resin. (**a**) Conductivity; (**b**) Seebeck coefficient; (**c**) ZT [93].

Since the thermoelectric performance of CFRC was first proposed, scholars at home and abroad have conducted generous research on it [85,86]. According to the thermoelectric merit ($\mathrm{ZT}=\left(S^{2}\sigma T\right)/K$), an excellent thermoelectric material should possess low thermal conductivity, high conductivity, and the Seebeck coefficient simultaneously. However, these parameters are interdependent, and an increase in conductivity may lead to an increase in thermal conductivity, so it is still a challenge to jointly optimize them to maximize their thermoelectric performance. Wen et al. [87] showed that the absolute thermoelectric power of original CFRC was only −0.8 μV/°C. Thus, many attempts were made to improve the thermoelectric performance of CFRC. The first was the selection of fiber type, including continuous fibers or cut fibers; P-type fibers or cut fibers directly affect the thermoelectric properties of CFRC in the direction of fiber [88]. The second improvement was the option of conductive filler, which mainly impacts the thermoelectric behavior of CFRC through the thickness direction. Metal compounds (such as Bi_2_Te_3_, Bi_2_O_3_, Bi_2_S_3_), conductive polymers (polycarbonate), and carbon nanomaterials (carbon nanotubes, carbon black) are commonly employed as interlayer conductive fillers. Wei et al. [89] first reported that the thermoelectric performance of CFRC could be effectively improved with the addition of 5.0 wt.% Bi_2_O_3_. The Seebeck coefficient could be increased to 4–5 times the original and the absolute thermal power up to 100.28 μV/°C. Likewise, the incorporation of Bi_2_Te_3_ particles could reduce the thermal conductivity and resistivity of CFRC [90]. Meanwhile, Jagadish et al. [91] obtained that 45 wt.% Bi_2_S_3_ had a significant advantage in collecting energy and converting it into electrical energy. Moreover, the effect of conductive polymers on thermoelectric performance should not be ignored. P_3_OT (poly (3-octyl thiophene)) itself has a high Seebeck coefficient [92]. The product obtained by compounding 50% P_3_OT with CFs had a Seebeck coefficient of 136 μV/°C and a maximum power factor of 7.05 μW m^−1^ K^−2^, which provided a new method for preparing CFRC with excellent thermoelectric performance. Wei et al. [93] treated CFs with different contents of phenolic resin and found that CFRC containing thin pyrolytic carbon layers exhibited typical semiconductor behavior, and its thermoelectric properties were comparable to those of oxide thermoelectric materials (Figure 9). Furthermore, extensive efforts have been made in investigating the influence of carbon nanomaterial conductive fillers for improving the thermoelectric properties of CFRC currently [76,94,95]. Cao et al. [95] observed that the incorporation of graphite significantly improved the Seebeck effect of CFRC, which the Seebeck coefficient aggrandized from 1.6 μV/°C to 16.1 μV/°C when the content of the graphite was 10 wt.%. Furthermore, the dominant conductivity type of CFRC gradually changed from the initial P-type conductivity to the N-type conductivity with the augmentation of the graphite content. It is clear that before the formation of the CF conductive network, the Seebeck coefficient gradually increases with increasing CF content. Then, the conductive network forms when the content of CFs is 0.6 wt.%. After that, carriers with positive electric charges randomly diffuse into the conductive network, resulting in the reduction in carrier concentration and Seebeck coefficient. Meanwhile, the incorporation of graphite increases the linearity and possibility of the Seebeck effect at this stage of CFRC. Additionally, the preparation process of CFRC is well related to electrical conductivity. Liu et al. [96] evaluated the influence of three different Bi_2_Te_3_ doping modes on the thermoelectric enhancement effect, namely, uniform volume mixing, gradient volume mixing, and gradient layer mixing. It was found that the thermoelectric energy of CFRC prepared by the gradient layer mixing process could reach 36.3 μV/°C, which was nearly 10 times higher than that of traditional ones. Furthermore, Wen et al. [97] discovered that the technology of bromine intercalation further enhanced the charge transfer between the intercalation layer and the carbon body in CFRC and increased the carrier concentration, which made the absolute thermal power reach −12 μV/°C.

By summarizing the research progress on thermoelectric performance in the past ten years (Table 4), it was found that compared with traditional CFRC, although the Seebeck coefficient and electrical conductivity have been greatly improved through a series of improved processes, they will inevitably lead to a thermal conductivity increase. Although the ZT value has been improved (ZT_max_ < 1), it invariably fails to meet the sensitivity requirements of satisfactory thermoelectric materials in the practical construction field. Further attempts and improvements are thereby required.

#### 5.3.2. Electrothermal Effect

**Figure 10:**
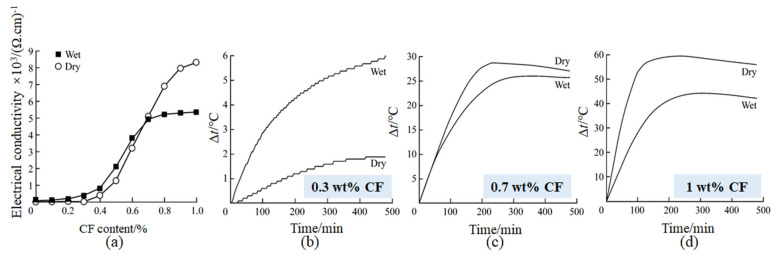
Variations in the (**a**) electrical conductivity and (**b**–**d**) electrothermal properties of CF content [101].

Apart from favorable conductivity and thermal conductivity, CFs with a disordered graphite structure manifest the advantages of a small diameter and large specific surface area, which make the Joule heat generated by energizing easy to emit. Their electrothermal conversion efficiency can also reach more than 90%. Although CFRC has been designed for various electric heating elements, there are still many drawbacks that need to be solved. For example, internal stress, unsaturated bonds, and impurities generally exist in CFs, as well as easy-to-produce oxidation weight loss when heated in an aerobic environment, resulting in an unstable electric heating performance of CFRC. Consequently, widespread attention has been paid to investigating the improvement and enhancement mechanism of the electrothermal properties of CFRC. The research of Hai et al. [101] concluded that the influence of moisture on the electrothermal effect is mainly reflected in conductivity. The moisture in the pore solution could promote the electrical conductivity and electrothermic effect at a low content of CFs (<0.3 wt.%). Conversely, the water film on the surface of CFs will increase the electronic transition resistance between CFs in the CFRC that has formed a conductive network (Figure 10). On this basis, Hai et al. [102] further found that the conductivity of CFRC prepared by the technique of a CF gradient distribution process was 131.8% higher than that of traditional concrete, while its maximum interlayer temperature difference was reduced to a varying degree. This principle provides a new consideration for reducing interlayer thermal stress. Chen et al. [103] modified the surface of polyacrylonitrile-based CFs with graphene/epoxy resin coating, which could increase the temperature of CFs by 40 °C, energizing at a voltage of 4 V for 60 s. The electrothermal properties also improved by about 21.4% and possessed good rapid temperature response sensitivity. Furthermore, it still had good electrothermal stability after 20 times of cold and hot cycles at a 6 V voltage. Lu et al. [10] designed a unique method of modifying aluminum surfaces based on siloxane groups and furtherly enhancing the electrothermal efficiency of CFs through chemical bonds. It is also a great choice to blend CFs with other conductive fillers to synergistically improve the thermoelectric properties of CFRC except for the modification of CFs. Xu et al. [104] stated that the incorporation of steel fibers at a low content of CFs (0.6–0.8 wt.%) was beneficial to the formation of the internal conductive network of CFRC, which significantly promoted the improvement of the electrothermal effect. When the content of CFs exceeded 1 wt.%, the incorporation of steel fibers would inhibit the homogeneous dispersion and effective connectivity of CFs, as well as air bubbles introduced, thereby reducing the electrothermal conversion rate. Wu et al. [105] found that conductive concrete mixed with CFs and carbon black can give full play to the advantages of the larger aspect ratio of CFs and short-range electrical conductivity of carbon black particles, making the resistivity as low as 1 Ω·m. In addition, Wang et al. [106] introduced a new modified mixing technology, in which the magnetically separated fly ash is used as conductive aggregate and then compounded with CFs. The conductive mortar prepared by this process not only has a high conductivity (<30 Ω∙cm), but also, the Seebeck coefficient is nearly 10 times higher than that of traditional CFRC (up to 2.63 mV/°C). It can be seen that the electrothermal performance of CFRC has a high application value in the field of intelligent sensing, which is worthwhile for further research [107].

#### 5.3.3. Strain-Sensitive Effect

The conductive network formed inside CFRC can respond quickly to external loads and environmental changes, showing a shocking strain sensitivity [108]. That is, electrical resistivity will decrease proportionally with increases in external mechanical strain, and conversely, it will increase gradually. These two effects of CFRC are reversible within an elastic range, and once the internal structure is damaged, electrical resistivity will change irreversibly [109]. It can usually be measured by the gauge factor (GF), which is defined as the ratio of the relative change in electrical resistance to mechanical strain, as shown in Formula (1):

(1) $$GF=\frac{\Delta R/R_{0}}{\Delta L/L_{0}}=\frac{\Delta R/R_{0}}{\varepsilon }$$

where, *GF*—strain-sensing sensitivity; Δ*R*—the value of resistance change; *R*_0_—initial resistance; Δ*L*—length change; *L*_0_—initial length; and *ε*—strain.

**Figure 11:**
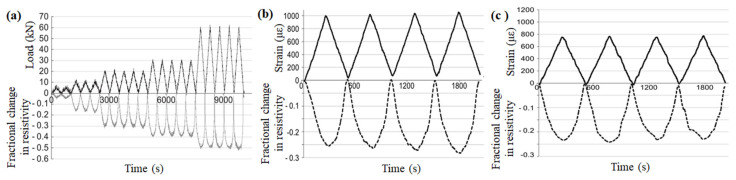
Response of CFRC under cyclic compressive loading at (**a**) different load amplitudes, (**b**) 15 wt.% CFs, and (**c**) 15 wt.% CFs + 1 wt.% CNT [111].

Fu et al. [110] first applied CFRC to monitoring the slight fatigue damage of the structure in 1996. It was found that the fatigue damage of CFRC incorporating 0.24 vol.% CFs will irretrievably make the electrical resistivity reduced by 2% in the first 10% fatigue life of compression or tensile strength, which might be caused by the bond separation between adjacent fibers. This phenomenon provides a new insight into the application of CFRC in the field of real-time structural health monitoring. To further improve the strain-sensing effect of CFRC, Azhari et al. [111] studied the resistivity response of CFRC under cyclic loading with different stress amplitudes (5, 10, 20, 30, 60 kN) (Figure 11a). On this basis, the strain response sensitivity of the conductive composites using CFs alone (Figure 11b) and a mixture of CFs and CNT (Figure 11c) were also compared, confirming that there was a good correspondence between the strain and fractional change in resistivity of CFRC, and the addition of carbon nanotubes could enhance the sensitivity of CFRC as well as having good repeatability under cycle compression loading. Xu et al. [112] placed CFRC under the action of a magnetic field and discussed the influence mechanism of the orientation and arrangement of internal fibers on piezoresistive sensing. The results show that CFRC arranged parallel to the magnetic field possesses superior sensitivity, repeatability, and stability in strain sensing. Moreover, the excellent strain-sensing behavior of CFRC is closely related to its inherent macro-properties. Thus, extensive attempts have been made to improve the strain-sensing effect through the technology of enhancement modification. Meanwhile, Fu et al. [39] reported a novel modification method for CFs, in which the CFs were first exposed to an atmosphere of ozone, and then used as conductive filler to prepare CFRC. It was concluded that CFRC modified by ozone not only enhanced the mechanical properties of the matrix but also significantly improved the sensitivity of electrical resistance to strain. Although some achievements have been made in improving the strain-sensing effect of CFRC, it should be pointed out that there is still much room for improvement, so more attention needs to be paid to future research. This has further stimulated researchers to improve the stress strain and damage monitoring ability of CFRC under static and dynamic loads through various measures.

So far, numerous studies have been carried out on the sensing response of electrical resistivity to the strain of CFRC under external actions, such as uniaxial compression, tension, bending, and repeated cyclic loading. The influence mechanism of CF content, loading amplitude, loading rate, and other factors on the sensitivity and stability of intelligent cement-based sensors has been well-defined, respectively. Various studies have confirmed that CFRC is a new type of intelligent cement-based material integrating structural functions and sensing functions. After calibration, it can not only be used for health monitoring and the condition evaluation of actual structures but also has good application potential in the fields of traffic monitoring, vehicle dynamic weighing, structural vibration control, and reinforcement corrosion condition monitoring [27,113].

## 6. Engineering Applications of CFRC

Owing to its remarkable characteristics of thermoelectric, electrothermal, and strain-sensing properties, CFRC could be well used to monitor the stress and strain state and crack damage in structures in real time, which shows a potential investment value in the areas of thermoelectric energy harvesting, deformation regulation, and structural health monitoring, as well as melting snow and ice and electromagnetic shielding.

### 6.1. Thermoelectric Energy Harvesting

**Figure 12:**
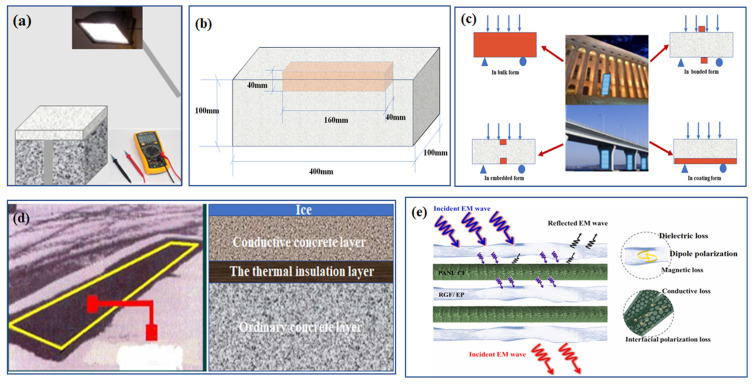
The application of CFRC in (**a**) Thermoelectric energy harvesting, (**b**) Intelligent adjustment, (**c**) Structural health monitoring, (**d**) Melting snow and ice, and (**e**) Electromagnetic shielding. ((**a**,**b**,**d**) are proposed by this paper while (**c**,**e**) are from Ref. [27] and Ref. [119], respectively).

Large cities often experience the heat island effect in hot summer, which is mainly caused by increases in the surface temperature of roofs and cement concrete pavements under the strong radiation of sunlight. Supposing the heat energy in the environment can be reasonably collected and converted into electric energy through conductive channels, this would play a good role in reducing the urban heat island effect. In recent years, apart from having a satisfactory structural function, the properties of CFRC can be altered for different applications compounded with various smart components. Thus, the emergence of thermoelectric cement-based materials has been tremendously promoted. The temperature difference induced by solar radiation triggers the CFRC for energy harvesting. To further explore its feasibility, Wei et al. [100] simulated the energy collection capacity of CFRC under solar radiation for the first time (Figure 12a). In the study, CFRC was made into a thin plate with a thickness of 20 mm and an area of 1 m^2^, and it was found that the energy obtained could reach 4–5 μW. This indicated that the ZT of CFRC is relatively low, resulting in a small amount of energy captured per square meter. Although this result is not very satisfactory, it is promising in shaping a great future. Future research should pay sufficient attention to the thermoelectric conversion efficiency of CFRC. On this basis, if the vast building surface of CFRC can be used reasonably and effectively for energy harvesting in summer, it will be an important effort that cannot be ignored for reducing worldwide energy consumption. Hence, CFRC with good thermoelectric conversion performance will have good application value in reducing the urban heat island effect, recycling waste heat in the construction industry, and thermoelectric energy harvesting.

### 6.2. Intelligent Adjustment of Structure

According to the unique characteristics of the electrothermal effect, the volume of self-adjusting smart concrete will expand during heating with electricity, which could be well used to realize the active control of the concrete structure. This function of self-adjusting smart concrete could alleviate the shortcomings of traditional concrete such as, for example, lower tensile strength, easy cracking, and sudden fracture without any symptoms, greatly facilitating the development of intelligent civil structures [10]. Although researchers have made great efforts to explore the potential of shape memory alloys in the deformation regulation of concrete beams, the high cost and compatibility with concrete are still the main factors restricting its popularization. However, CFRC equipped with good electrothermal properties has obvious advantages in having a fast heating rate and ease of control, which could be considered to be embedded into concrete to achieve structural deformation adjustment. Yao et al. [114] designed a new structure combination mode, in which the CFRC with a superior electrothermal effect was embedded in the upper part of the concrete beam and then heated with electricity (Figure 12b). This method is equivalent to applying compressive pre-stress to the bottom layer of the concrete beam by using the volume expansion generated by the electric heating of CFRC, such that the bearing capacity of the concrete beam can be greatly improved when it reaches the same downward deflection. The results showed that under the control of CFRC deformation regulation, the peak load of concrete beams could be increased by more than 50%, which deeply facilitates the wide application of CFRC to realize low-cost and intelligent structural deformation adjustment in the field of civil engineering in the future.

### 6.3. Structural Health Monitoring

CFRC possesses a significant stress strain-sensing effect, which can be considered as a sensor embedded into beams, columns, piers, and other components to form intelligent systems for the application of structural health monitoring in civil engineering [17]. This system is a non-destructive monitoring system, and it mainly monitors and evaluates the stress and strain state and crack damage degree in real time through data testing to make a rapid response. Generally, the self-monitoring concrete prepared by CFs (0.2–0.5 vol.%) is endowed with a reversible stress and strain-sensing effect. It should be noted that an increase in reversible resistivity provides an indication of reversible strain with a strain-sensitive factor of up to 700 [113]. Moreover, the structural form of CFRC as a sensor also tends to diversify with the development of science and technology, which is transformed from the bulk gradually into the coating, interlayer, bonding, and embedding. The detailed structural arrangement is shown in Figure 12c [27]. The commonly used sensor mainly comprises letting the CFRC smart cement-based blocks with a size of aggregate embed into concrete structures. Compared with other forms, it not only has high sensitivity and good compatibility but can also realize high-efficiency, large-scale, and distributed intelligent monitoring of infrastructure due to its cost advantage [115]. Moreover, the smart sensor prepared by adding conductive filler likewise has good bending strength and toughness, showing outstanding advantages in optimizing structural design and building smart cities.

### 6.4. Melting Snow and Ice

Traditional snow and ice removal techniques mainly include the use of deicing salt and mechanical removal, which not only affects the durability of the structure but also consumes huge financial and material resources. According to statistics, the US, China, and the European Union spend nearly 6, 10, and 9 billion dollars, respectively, on dealing with snow and ice accumulation on roads each year. To overcome the shortcomings of the existing technology, researchers have found that incorporating appropriate amounts of CFs into cement-based materials can significantly improve the electrical conductivity of the structures. Based on the inherent resistivity of the CFRC to current flow, heat can be generated by applying an electric potential to the CFRC using embedded electrodes. Moreover, the electrothermal conversion efficiency of CFRC is extremely high, so it can be considered for applying the Joule heat generated by energizing the CFRC to the snow melting and deicing of airport runways in winter (Figure 12d). The application advantages of CFRC are mainly reflected in the automation of road maintenance operations in winter and reducing the use of hazardous deicing chemicals, thus greatly increasing the service life of road structures. To further explore the feasibility of CFRC alternatives to traditional schemes for heated pavement systems, Sassani et al. [116] investigated the percolation characteristics of CFs in different carriers and the electrothermal properties of CFRC under long-term experimental tests. The results show that CFs can reach the percolation threshold of CFRC at a volume dosing of 0.25–1%, 0.6–1%, and 0.5–0.75% in paste, mortar, and concrete, respectively. Furthermore, the resistivity of CFRC from 28 d to 460 d decreases from 1.86 × 10^−2^ to 1.22 × 10^−2^ S/cm, and the corresponding electrothermal conversion efficiency decreases by only 16%. More importantly, this suggests that the optimal CF dosage of CFRC with a stable electrothermal effect in a long-term service environment should be controlled at 1 vol.%, which provides a certain reference value for realizing low energy consumption and high-efficiency snow and ice melting in large areas of pavements.

### 6.5. Electromagnetic Shielding

CFRC itself has good electrical conductivity, which can effectively absorb, reflect, and shield the electromagnetic wave pollution and leakage caused by radio communication (Figure 12e). It was found that the shielding effect of ordinary concrete under 500 Hz radiation is 1 dB, while the incorporation of 3 vol.% CFs can enhance its shielding effect by 15 times. To further improve the electromagnetic shielding effect of CFRC in smart city applications, Xie et al. [117] found that compounding CFs with iron nanoparticles had a higher shielding effect than using CFs as the only filler, with a shielding value of 70 dB at 0.1 GH radiation. Additionally, the surface chemistry of CFs is an important parameter to measure the interaction between the cement matrix and the fibers. The surface pretreatment of CFs utilizing heat treatment or chemical agents can increase relative surface area, activate the surface chemical functional groups, and effectively improve the degree of dispersion and bonding strength with the matrix. The chemical vapor deposition technique is based on the surface structural changes caused by the pyrolytic carbon deposition of CF at 1200 °C to enhance the electromagnetic shielding effect of CFRC. Meanwhile, Fu et al. [118] improved the electromagnetic shielding performance of CFRC by chemically plating a thin layer of nickel on the CF surface to enhance the interfacial bonding with the matrix, and the shielding value could be as high as 87 dB at 1 GHz radiation. The existing modification techniques have demonstrated the potential application of CFRC in the field of electromagnetic shielding, among which the combination of nickel and CFs as one of the most effective ways to enhance the shielding effect of CFRC at present.

## 7. Conclusions and Future Research Needs

With the rapid increase in the production of domestic CFs, the problem of higher preparation costs of CFRC has been gradually alleviated. Compared with traditional cement-based materials, CFRC possesses the characteristics of high strength and durability, good electrical conductivity, and sensitivity to temperature and stress. Based on those characteristics, CFRC could be considered to be made as an intelligent system integrating mechanical and sensing properties, which greatly promotes the application potential of CFRC in the fields of real-time health monitoring, thermoelectric energy harvesting, structural intelligent regulation, and so on. To develop a CFRC with excellent properties, the preparation process, conductive transmission mechanism, and mechanical and smart properties under the actual service environment are key factors affecting its performance. Hence, aiming at the challenge of homogeneous dispersion of hydrophobic CFs in the cement matrix, this paper first provides state-of-the-art information on the CFRC reinforcement modification mechanism and CF dispersion evaluation method. The conductive transmission mechanism of CFRC and the improvement effect of different strengthening mechanisms on the mechanical properties, durability, and smart properties of CFRC are also deeply discussed. The main conclusions and relevant suggestions are as follows:

(1) Due to the strong van der Waals forces and surface hydrophobicity between CFs, the main challenge facing the preparation process of CFRC is how to improve the uniform dispersion of CFs in the cement slurry and enhance the interfacial bond strength between CFs and the cement matrix. Although the dispersion degree of CFs has been greatly improved through a series of physical dispersion and chemical modification methods, the preparation process is relatively complicated and cannot be used in practical engineering structures on a large scale. Further research could consider developing some useful but uncomplicated stirring processes to prepare CFRC and simultaneous multi-scale compounding with other functional fillers to further improve the mechanical and sensing properties of CFRC.

(2) The homogeneous distribution of CFs in the cement matrix is the foundation of the functionality of CFRC. The evaluation of the dispersion degree of CFs is mainly based on the macroanalysis of SEM images or lateral evaluation through electrical and mechanical properties in recent years. The area observed by scanning electron microscopy is limited and not representative. Meanwhile, the results obtained by electrical and mechanical property tests are closely related to the moisture content of the sample itself and the bonding conditions between the sample and the electrode, making it difficult to accurately assess the dispersion of CFs within CFRC. Therefore, an urgent problem is to establish a set of evaluation systems to guide and regulate the dispersion quality of CFs in the matrix.

(3) The incorporation of CF can give full play to the toughening effect in the matrix, and its working principle is mainly manifested in changing the cracking path of microcracks, blocking the cracking path, and playing the bridging role of cement paste. In general, 0.5% and 1% are considered to be the optimal volume dosages of CFRC to ensure good mechanical properties and maintain large workability. This is because the high volume fraction of CFs will cause inherent defects within the CFRC, which will greatly reduce the performance. However, in terms of smart sensing, the strain coefficient decreased from 1.16 to 0.6 when the content of CFs increased from 0.1 vol.% to 1.5 vol.%, indicating that CFRC with low CF dosage has a higher degree of strain response. Based on the comprehensive consideration of the influence of environmental factors such as temperature and humidity, it is recommended that the optimal dosing of CFRC be set in the range of 0.5–1.5 vol.%. CFRC is equipped with satisfactory smart properties (thermoelectric, electrothermal, and strain-sensitive effect) while being relatively insensitive to environmental factors. If properly calibrated, CFRC can realize cost-effective adjustment and monitoring of structures without other auxiliary sensing devices.

(4) The scattered CFs in concrete can fully exert the bridging effect to effectively inhibit the generation of connected cracks, such that CFRC shows great durability in environments of freezing and thawing, chloride ion penetration, carbonization, and sulfate corrosion. However, most of the current research is based on the discussion of the durability of CFRC under the action of a single action, while the research on the degradation law of the durability and smart properties of CFRC is still very insufficient; so, further research is needed to guide the durability design of CFRC in the actual service environment.

(5) There is a good corresponding relationship between the resistivity change and its stress strain state of CFRC. The improvement of mechanical properties, durability, and smart properties of CFRC is still limited by the factors of CF content, length, and dispersion degree. Thus, adjusting the interaction behavior between CFs and the cement matrix by an appropriate enhancement modification mechanism is required. The integration of intelligent structures can be realized through early warning, adaptive adjustment, and self-repair to improve the safety and reliability of CFRC. This is of great significance for further research and application of CFRCs in the fields of structural real-time health monitoring, thermal energy harvesting, intelligent deformation adjustment, and other engineering fields.

## Figures and Tables

**Table 1:** Mechanical and durability properties of unmodified CFRC.

Properties	Types	w/b Ratio	Content of CFs	Modification	Enhancement in Properties	References
Compressive strength	Concrete	–	0.55 vol.%	No	8.3%	[30]
Flexural strength	Concrete	–	1 vol.%	No	20%	
Compressive strength	Mortar	0.51	3 vol.%	No	61.5%	[61]
Bending strength	Mortar	0.51	4 vol.%	No	74.5%	
Splitting strength	Mortar	0.51	4 vol.%	No	58.1%	
Compressive strength	Concrete	0.46	0.2 vol.%	No	3.8%	[78]
Flexural strength	Mortar	0.35	1 vol.%	No	36.3%	[79]

**Table 2:** Mechanical and durability properties of physically modified CFRC.

Properties	Types	w/b Ratio	Content of CFs	Modification	Enhancement in Properties	References
Compressive strength	Concrete	0.4	0.2 vol.%	Hybrid with 0.3 vol.% steel fibers	31.4%	[62]
Splitting tensile strength	Concrete	0.4	0.2 vol.%	Hybrid with 0.3 vol.% steel fibers	36.5%	
Modulus of rupture	Concrete	0.4	0.2 vol.%	Hybrid with 0.3 vol.% steel fibers	32.9%	
Flexural toughness	Concrete	0.4	0.2 vol.%	Hybrid with 0.3 vol.% steel fibers	33.9–199.5%	
Compressive strength	Recycled concrete	0.41	0.5 vol.%	Addition of silica fume	20%	[74]
Tensile strength	Recycled concrete	0.41	0.5 vol.%	Addition of silica fume	34%	
Flexural strength	Recycled concrete	0.41	0.5 vol.%	Addition of silica fume	46%	
Acid attack resistance	Recycled concrete	0.41	0.5 vol.%	Addition of silica fume	40%	
Compressive strength	Concrete	0.46	0.2 vol.%	Hybrid with 1 vol.% steel fibers	8%	[78]
Compressive strength	Mortar	0.4	0.75 vol.%	Addition of bottom ash	18.75%	[80]
Bending strength	Mortar	0.4	0.75 vol.%	Addition of bottom ash	11.1%	
Flexural strength	Mortar	0.5	0.4 wt.%	Carbon nanotubes (CNTs)-nano-SiO_2_ (NS) modified CFs	14.1%	[81]
Compressive strength	Mortar	0.5	0.4 wt.%	CNTs-NS modified CFs	10.18%	
Autogenous shrinkage	Mortar	0.5	0.4 wt.%	CNTs-NS modified CFs	27%	
Dry shrinkage	Mortar	0.5	0.4 wt.%	CNTs-NS modified CFs	14.8%	
Impermeability	Mortar	0.5	0.4 wt.%	CNTs-NS modified CFs	15.1%	

**Table 3:** Mechanical and durability properties of chemically modified CFRC.

Properties	Types	w/b Ratio	Content of CFs	Modification	Enhancement in Properties	References
Compressive strength	Mortar	0.45	0.5 wt.%	0.3% hydroxypropyl cellulose dispersed CFs	14.28%	[29]
Compressive strength	Mortar	0.45	0.5 wt.%	0.5% polyvinyl pyrrolidone dispersed CFs	17.6%	
Bending strength	Mortar	0.45	0.5 wt.%	0.3% hydroxypropyl cellulose dispersed CFs	1.28%	
Bending strength	Mortar	0.45	0.5 wt.%	0.5% polyvinyl pyrrolidone dispersed CFs	15.38%	
Tensile strength	Mortar	0.32	0.51 vol.%	O_3_ modification	38%	[40]
Tensile modulus	Mortar	0.35	0.51 vol.%	O_3_ modification	82%	
Ductility	Mortar	0.23	0.51 vol.%	O_3_ modification	26%	
Compressive strength	Mortar	0.39	0.6 wt.%	Dispersant and ultrasound acting together	20%	[59]
Tensile strength	Mortar	0.39	0.6 wt.%	Dispersant and ultrasound acting together	140%	
Modulus	Mortar	0.39	0.6 wt.%	Dispersant and ultrasound acting together	26.8%	
Bending strength	Mortar	0.5	1.5 vol.%	SiO_2_ coated CFs	10.4% (compared with uncoated specimen); 58.1% (compared with the plain)	[60]

**Table 4:** Thermoelectric properties of CFRC.

Types	S (μV/°C)	ρ (Ω cm)	Thermoelectric Power (μV/K)	Electrical Conductivity (S/m)	Thermal Conductivity (W/(m·K)	ZT	References
0.4 wt.% CFs	7 ± 0.3	(2.8 ± 0.2) × 10^3^	8.8 ± 0.3				[76]
0.4 wt.% CFs + 0.5 wt.% CNTs	21.7 ± 3.6	(7.1 ± 0.2) × 10^2^	23.5 ± 3.6				
1 wt.% CFs	−2.82 ± 0.11	(8.3 ± 0.5) × 10^2^	−0.48 ± 0.11				[82]
Normal lightweight concrete with CFs	125.1		127.44				[83]
Lightweight concrete with fly ash and CFs	65.3		67.64				
Lightweight concrete with silica fume and CFs	49.7		52				
Pristine CFs	−3.13 ± 0.16		−0.79 ± 0.16				[87]
Bromine intercalated CFs	−18.9 ± 1.32		−16.6 ± 1.32				
5 wt.% Bi_2_O_3_ + CFs	97.94		100.28				[89]
CF epoxy polymer (Te + Bi_2_Te_3_ + carbon black)	–	0.02	163		0.51	0.09	[90]
50 wt.% CFs	136			380			[92]
Thin pyrolytic carbon layer modified CFRC						3.11 × 10^−3^	[93]
3.0 wt.% Ca_3_Co_4_O_9_ modified CFRC	58.6						[98]
1 wt.% CFs + CeFe_4_Sb_12_	89.9			2.01 × 10^5^	2.45	0.9 (800 K)	[99]
1 wt.% CFs	19.73		22.07	0.2008	0.22	27 °C: 1.334 × 10^−7^ 89 °C: 1.609 × 10^−7^	[100]

## Data Availability

The study did not report any data.
